# The Impact of Neck Pain on Work Productivity and Quality of Life Among Office Workers in Jazan: A Cross-Sectional Study

**DOI:** 10.7759/cureus.76254

**Published:** 2024-12-23

**Authors:** Mohammed H Alhazmi, Zenat Khired, Mohammed A Alhazmi, Basem Zogel, Hussam Darraj, Sultan M Hakami, Salman M Qahl, Khalid M Hakami, Omar Hadadi, Osama A Mobarki

**Affiliations:** 1 General Medicine, Damad General Hospital, Jazan, SAU; 2 Surgery, Jazan University, Jazan, SAU; 3 Orthopedics and Trauma, Sabya General Hospital, Jazan, SAU; 4 General Medicine, Abu Arish General Hospital, Jazan, SAU; 5 General Medicine, Jazan General Hospital, Jazan, SAU; 6 Orthopedic Surgery, Aseer Central Hospital, Abha, SAU; 7 General Medicine, Ministry of Health, Jazan, SAU

**Keywords:** neck pain, office workers, productivity, qaly, saudi arabia

## Abstract

Background and objective

Neck pain (NP) is a prevalent musculoskeletal disorder (MSD) among office workers (OWs) and significantly affects productivity and quality of life (QALY). However, the effect of NP on office employees in Saudi Arabia remains unclear. In light of this, we aimed to evaluate the impact of NP and its associated factors on OWs in the Jazan region, Saudi Arabia.

Methods

This cross-sectional study employed an anonymous, self-administered online questionnaire distributed to OWs (n=371) at Jazan University. The survey included demographic information, neck problem assessment, and the World Health Organization Quality-of-Life Scale Brief Version (WHOQOL-BREF) and Standardized Nordic questionnaire for those reporting NP. Descriptive statistics and logistic regression were used to examine the relationships between the variables and NP duration.

Results

The cohort comprised 221 (59.6%) males and 150 (40.4%) females, with a mean age of 37.61 years [standard deviation (SD): 11.04)]. The majority (360, 97.0%) were Saudi nationals, and 255 (68.7%) held a bachelor's degree. A substantial proportion of participants (60, 16.4%) reported missing work due to NP the previous year; however, only 97 (26.1%) had sought medical attention. NP significantly affected life satisfaction and QALY, with only 84 (22.6%) and 74 (12.5%) of the respondents reporting high satisfaction with their health and sleep, respectively. Regarding work impact, 42 (11.3%) reported NP affecting work for one to seven days in the past year, 13 (3.5%) for 8-30 days, and six (1.6%) for >30 days but not every day. Logistic regression analysis revealed that individuals without a history of neck accidents had a 48% lower likelihood of experiencing prolonged NP (8-30 days) in the past year [odds ratio (OR)=0.528, 95% confidence interval (CI): 0.279-0.999, p=0.050]. No significant associations were found between NP duration and factors such as age, sex, smoking status, or working hours (p>0.05).

Conclusions

This study elucidated the significant impact of NP on work productivity and QALY among OWs in Jazan, Saudi Arabia. Despite its high prevalence, the low rate of medical consultation sought suggests potential barriers to healthcare utilization. These findings underscore the necessity for tailored workplace interventions, improved access to medical services, and further research to address this occupational health concern and enhance the overall well-being of OWs.

## Introduction

Musculoskeletal disorders (MSDs) constitute a significant cause of disability worldwide and affect a substantial proportion of the workforce. Neck pain (NP), a common manifestation of MSDs, is a major cause of morbidity and functional impairments. It significantly impacts the daily lives and activities of approximately 34.4% of office workers (OWs) worldwide annually [1,2]. NP is a multifaceted condition with numerous contributing factors, including an individual's functional capacity, physical activity level, psychosocial status, and occupational habits. The prevalence of NP is higher in Asia than in Western countries, suggesting the influence of additional factors such as lifestyle and ethnicity. The burden of NP is increasing, particularly in low- and middle-income nations, significantly impacting individuals' quality of life (QALY) and placing strains on healthcare systems [1-4]. This trend underscores the urgent need for effective interventional strategies.

In most cases of NP, the challenge lies in the lack of a specific, identifiable etiology (e.g., a fracture), highlighting the complexity of this condition and the need for further research. Several modifiable and non-modifiable factors may contribute, individually or in combination, to the development of NP in nonspecific cases. These primarily include biological and personal risk factors such as advanced age, female sex, physical inactivity, low capacity of the neck and shoulder muscles (i.e., neck flexor endurance) [5-8], self-perceived high muscle tension [9], poor neck and head posture [2], reduced range of motion (e.g., of the cervical spine) [10], personal traits [3], and individual behavior [11]. Some studies have suggested that the onset of neck discomfort may have a genetic component [11]. Secondly, pain-related behavior [11,12], pain catastrophizing [11,13], fear-avoidance beliefs [11,13], and work-related stress [7,12] may all contribute to neck discomfort in OWs.

Women have been reported to exhibit higher personal pressures but fewer job stressors than men. Additionally, several factors contribute to NP in OWs, including sedentary work [6], low task variation, extended periods of computer use [10], high job demands [14], job insecurity [15], occupying a leadership position, suboptimal workplace environments, and ergonomic demands [16]. Furthermore, additional risk factors have been identified, such as sleep quality and social support [16]. Notably, the risk factors for NP are largely similar to those associated with reduced job productivity due to NP [17].

The volume of research in this field is increasing, and researchers are trying to identify the most effective therapies to assist patients with NP and to reduce the need for surgical interventions. Various treatments, such as active breaks and postural adjustments, have been investigated and proven effective in relieving NP in OWs [18]. As the number of individuals at risk for NP is projected to increase over the next decade [1], appropriate care strategies for treating and preventing NP are essential. A more comprehensive understanding of the causal relationship between NP and the risk factors that may affect individuals in specific settings is required to gain further insights into this issue. Therefore, this study aimed to evaluate the impact of NP and its associated factors among OWs at Jazan University in Saudi Arabia. We believe the findings will assist governmental administrations in understanding this situation better, enabling them to provide improved conditions for affected individuals.

## Materials and methods

Study design and setting

This cross-sectional observational study surveyed university OWs in Jazan, Saudi Arabia.

Study population and sampling

The study population comprised OWs in the Jazan region. Eligible participants had to be over 18 years of age and had experienced cervical pain of any intensity. The exclusion criteria included pregnancy, active infections, hospitalization, and lumbar discomfort. We used the Raosoft calculator (Raosoft Inc., Seattle, WA) to determine the required sample size of 377 participants, assuming a 50% response distribution, 95% confidence interval (CI), and 5% margin of error.

Data collection

Convenience sampling was employed to distribute an anonymous, online questionnaire via social media platforms, including Telegram, Twitter, and WhatsApp, for data collection purposes. The questionnaire, comprising three sections, was adapted from previously validated instruments [15-21]. It involved the following sections: (1) demographic information: age, sex, height, weight, nationality, education, marital status, smoking habits, employment, and weekly work hours; (2) neck problem assessment: a single question to evaluate NP, discomfort, or aches and (3) for respondents reporting NP, the World Health Organization Quality-of-Life Scale Brief Version (WHOQOL-BREF) questionnaire [21] and the Standardized Nordic questionnaire [19-20] were administered.

Pilot study

A pilot study was conducted with 20 participants to evaluate the questionnaire’s accuracy, language clarity, and completion time. Feedback from the participants was carefully reviewed, identifying issues related to question phrasing and cultural relevance. Based on this feedback, the following actions were taken: (1) ambiguous questions were rephrased for improved clarity; (2) terms were adjusted to ensure cultural appropriateness; and (3) the completion time of the questionnaire was shortened to ensure feasibility and reduce participant fatigue. These modifications ensured the final questionnaire was both user-friendly and effective for data collection.

Statistical analysis

Statistical analysis of the data was performed using IBM SPSS version 26 (IBM Corp., Armonk, NY). We obtained a comprehensive overview of the sample characteristics by computing the descriptive statistics. This included dispersion [standard deviation (SD)] and central tendency (mean) measurements for continuous age, height, and weight variables. Frequencies and percentages were calculated to analyze the distribution within the sample for categorical variables, such as gender, education level, and marital status. A comprehensive logistic regression analysis was conducted to examine the relationships between the variables, focusing on components related to the duration of neck discomfort.

This advanced statistical method facilitated the investigation of the impact of various factors, including work-related characteristics, lifestyle choices, and demographic factors, on the likelihood of experiencing neck discomfort for different durations over the past year. The logistic regression model considered four different durations of neck discomfort using a baseline of one to seven days for comparison. This approach enabled the measurement of correlations between the potential risk factors and extended episodes of neck discomfort. This analysis provides valuable insights into complex interactions among the different factors that affect the neck health of OWs. A p-value <0.05 was considered statistically significant.

Ethical considerations

The Jazan University Research Ethics Committee (REC) approved the study protocol. All individuals provided informed consent before participation. Participants were guaranteed the freedom to leave the study at any time without facing repercussions, and the privacy of their data was ensured.

## Results

Population characteristics

In total, 371 individuals were eligible to participate in this study. Of them, 360 (97.0%) were Saudi, and 11 (3.0%) were non-Saudi. The sample comprised 221 (59.6%) males and 150 (40.4%) females. The mean age was 37.61 years (SD: 11.04; range: 18-73 years). The mean height was 163.6 cm (SD: 16.8 cm), while the mean weight was 73.5 kg (SD: 17.8 kg). Sixty-nine (18.6%) participants were smokers. Smoking was more prevalent among males than females (26.2% vs. 7.3%, respectively). While most respondents were married (247, 66%), 108 (29.1%) were single, 12 (3.2%) were divorced, and four (1.1%) were widowed. Regarding the level of education, the majority had a bachelor's degree (255, 68.7%), 40 (10.8%) had only completed secondary school, 53 (14.3%) had a diploma, 17 (4%) had a master’s degree, and six (1.6%) had a doctorate. As for the duration of work, the majority 298 (80.3%) had eight-hour-long working duties, 10 (2.7%) had 24-hour-long working duties, 34 (9.2%) had 12-hour-long working duties, and 29 (7.8%) had four-hour-long working duties (Table 1).

**Table 1:** Characteristics of the study population SD: standard deviation

Variables	Values
Gender, n (%)	
Male	221 (59.6%)
Female	150 (40.4%)
Age, years, mean (SD)	37.61 (11.04)
Height, cm, mean (SD)	163.6 (16.8)
Weight, kg, mean (SD)	73.5 (17.8)
Smoker, n (%)	
Yes	69 (18.6%)
No	302 (81.4%)
Nationality, n (%)	
Saudi	360 (97.0%)
Non-Saudi	11 (3.0%)
Education level, n (%)	
Secondary	40 (10.8%)
Diploma	53 (14.3%)
Bachelor's degree	255 (68.7%)
Master's degree	17 (4.6%)
Doctorate	6 (1.6%)
Marital status, n (%)	
Married	247 (66.6%)
Single	108 (29.1%)
Divorced	12 (3.2%)
Widowed	4 (1.1%)
Duration of duty at work, n (%)	
24 hours	10 (2.7%)
12 hours	34 (9.2%)
8 hours	298 (80.3%)
4 hours	29 (7.8%)

Impact of neck pain on the productivity of office workers

In response to the question of whether neck trouble had prevented them from doing their normal work, 310 (83.6%) reported that they had never stopped working, 42 (11.3%) reported that it only prevented them from working for one to seven days in the last 12 months, 13 (3.5%) reported that it stopped them working for about 8-30 days, and six (1.6%) reported that it stopped them working for >39 days, but not every day. Furthermore, only 97 (26.1%) respondents had seen a medical practitioner for their NP. When asked if they needed medical treatment, 199 (53.6%) said that they did not require any medical treatment to function, 90 (24.3%) required it in small amounts, 52 (14.0%) required it in moderate amounts, 18 (4.9%) required a great deal of it, and 12 (3.2%) needed an extreme amount of medical treatment to function (Table 2).

**Table 2:** Effects of neck pain on productivity

Statement/query	Response	N	(%)
The duration that neck trouble has prevented you from doing normal work	Never stopped working	310	83.6
	1-7 days	42	11.3
	8-30 days	13	3.5
	>30 days but not every day	6	1.6
Has physical pain prevented you from doing what you need to do?	Not at all	69	18.6
	Small amount	117	31.5
	Moderate amount	109	29.4
	Great deal	43	11.6
	Extreme amount	33	8.9
Have you seen a medical practitioner due to neck problems?	Yes	97	26.1
	No	274	73.9
How much medical treatment do you need to function?	Not at all	199	53.6
	Small amount	90	24.3
	Moderate amount	52	14
	Great deal	18	4.9
	Extreme amount	12	3.2

Impact of neck pain on quality of life and life satisfaction

In response to the question about their level of satisfaction with themselves, only 127 (34.2%) of the respondents were very satisfied, while 127 (43.9%) were satisfied with their lives, 54 (14.6%) were neither dissatisfied nor satisfied, 20 (5.4%) were fairly dissatisfied, and seven (1.95%) were very dissatisfied. Furthermore, 84 (22.6%) participants were very satisfied with their health, 174 (46.9%) were satisfied, 57 (15.4%) were neither dissatisfied nor satisfied, 40 (10.8%) were fairly dissatisfied, and 16 (4.3%) were very dissatisfied with their health. In contrast, 47 (12.5%) of the respondents were very satisfied with their sleep, 130 (35%) were satisfied, 96 (25.9%) were neither dissatisfied nor satisfied, 65 (17.5%) were fairly dissatisfied, and 33 (8.9%) were very dissatisfied. As for their living conditions, 84 (22.6%) of the participants were very satisfied, 192 (51.8%) were satisfied, 65 (17.5%) were neither dissatisfied nor satisfied, 22 (5.95%) were fairly dissatisfied, and 8 (2.2%) were very dissatisfied (Table 3).

**Table 3:** Impact of neck pain on quality of life and life satisfaction

Query	Response	N	(%)
How satisfied are you with your health?	Very dissatisfied	16	4.3
	Fairly dissatisfied	40	10.8
	Neither satisfied nor dissatisfied	57	15.4
	Satisfied	174	46.9
	Very satisfied	84	22.6
How satisfied are you with your sleep?	Very dissatisfied	33	8.9
	Fairly dissatisfied	65	17.5
	Neither satisfied nor dissatisfied	96	25.9
	Satisfied	130	35
	Very satisfied	47	12.7
How satisfied are you with yourself?	Very dissatisfied	7	1.9
	Fairly dissatisfied	20	5.4
	Neither satisfied nor dissatisfied	54	14.6
	Satisfied	163	43.9
	Very satisfied	127	34.2
How satisfied are you with your living conditions?	Very dissatisfied	8	2.2
	Fairly dissatisfied	22	5.9
	Neither satisfied nor dissatisfied	65	17.5
	Satisfied	192	51.8
	Very satisfied	84	22.6

Factors associated with the duration of neck pain among office workers in the past year

Logistic regression was conducted to ascertain the effect of certain demographic factors, such as age and gender; work-dependent factors, such as working duties; and lifestyle-related factors, such as smoking and a history of accidents; on the number of days the participants reported NP in the past 12 months. The logistic regression model was not statistically significant: 0.57. Four categories of NP duration were considered, with "one to seven days" as a baseline. The regression analysis model found that people with no history of neck accidents had a 48% reduction in their chance of experiencing NP lasting 8-30 days in the past year. None of the other factors were significantly associated with the likelihood of NP lasting more than seven days in the past year (Table 4).

**Table 4:** Logistic regression analysis of the predictors of neck pain duration among office workers CI: confidence interval; OR: odds ratio

Category	Variable	B	Significance	OR	95% CI	
8-30 days	Intercept	2.722	0.408			
	Gender	-0.101	0.806	0.904	0.405	2.017
	Smoker	-0.620	0.106	0.538	0.254	1.141
	Working duty	-0.099	0.738	0.906	0.506	1.621
	Have you ever had a neck accident?	-0.639	0.050	0.528	0.279	0.999
	Age, years	-0.005	0.751	0.995	0.967	1.024
	Height, cm	-0.009	0.562	0.991	0.961	1.022
30 days, but not every day	Intercept	-0.484	0.893			
	Gender	0.176	0.651	1.192	0.557	2.549
	Smoker	0.014	0.973	1.014	0.456	2.256
	Working duty	-0.085	0.755	0.919	0.539	1.567
	Have you ever had a neck accident?	-0.268	0.364	0.765	0.428	1.365
	Age, years	-0.023	0.096	0.978	0.952	1.004
	Height, cm	0.003	0.880	1.003	0.968	1.038
Everyday	Intercept	-6.386	0.152			
	Gender	0.575	0.211	1.777	0.722	4.374
	Smoker	-0.502	0.242	0.605	0.261	1.403
	Working duty	0.073	0.830	1.076	0.553	2.093
	Have you ever had a neck accident?	0.155	0.650	1.167	0.598	2.276
	Age, years	0.018	0.255	1.018	0.987	1.049
	Height, cm	0.023	0.298	1.023	0.980	1.068

## Discussion

The current study describes the causes, consequences, and risk factors of neck discomfort among OWs in Jazan. According to our research, this demographic experiences a significant burden of neck discomfort, substantially impacting QALY, work productivity, and healthcare utilization patterns.

Impact on work

According to our findings, 16.4% of participants reported missing work in the preceding year due to neck discomfort, with 11.3% missing one to seven days, 3.5% missing 8 to 30 days, and 1.6% missing more than 30 days but not every day. These results align with other research emphasizing neck discomfort's significant negative influence on workplace productivity. Hoy et al. found that the yearly prevalence of neck discomfort that limits one's activities ranged from 7.5% to 14.5% [4]. The somewhat higher frequency in our study population may be attributed to the specific work environment of OWs.

Healthcare utilization

Analyzing the low rate of medical consultations in Jazan, our study found that only 26.1% of participants sought medical attention for neck pain. This is consistent with findings by Côté et al.'s study, where 31.4% of individuals with neck pain in the United States consulted a physician [15,22]. These similarities suggest potential barriers to healthcare utilization that may extend beyond regional differences.

Impact on the quality of life

Our research indicated that neck discomfort significantly diminishes life satisfaction and QALY. Only 12.5% of participants reported high satisfaction with their sleep, while only 22.6% expressed high satisfaction with their health. These findings corroborate those of earlier studies that emphasized the detrimental impact of neck discomfort on overall health. Salo et al. demonstrated that chronic neck discomfort was associated with reduced health-related QALY, particularly in physical pain and functioning domains [23].

Associated factors

According to the logistic regression study, the probability of experiencing chronic NP (8-30 days) in the past year was 48% lower in individuals without a history of neck injury. This study underscores the importance of neck injury prevention and suggests that prior trauma may be a significant risk factor for persistent or recurrent neck discomfort. Similar associations have been reported in the literature; a review identified a history of neck injury as a predictor of poor outcomes in patients with neck discomfort [15-24]. Our study found no significant correlations between neck discomfort duration and age, sex, smoking status, or working hours, which contrasts with several studies that identified these as potential risk factors. For instance, Cagnie et al. (2007) found that older age and female gender were associated with a higher prevalence of neck discomfort in OWs [6]. The absence of such associations in our study may be attributed to the specific characteristics of our sample or the cultural factors unique to the Saudi Arabian context.

Workplace interventions and ergonomics

The high prevalence of neck discomfort in our study population emphasizes the need for effective workplace intervention. Although ergonomic factors were not specifically assessed in our study, previous research has indicated that prolonged static postures and poorly designed workstations significantly contribute to neck discomfort among OWs. A systematic review by Jun et al. (2017) identified prolonged computer use and awkward postures as physical risk factors for neck discomfort in OWs [9].

Psychosocial factors

While our study did not explicitly examine psychosocial factors, their impact on life satisfaction and QALY suggests their relevance in the experience of NP. Previous studies have demonstrated the importance of psychological factors in the onset and persistence of NP. Yang et al. found an association between neck discomfort in US workers and workplace psychosocial factors such as job demands and job insecurity [15]. Future research in this population should include a more comprehensive assessment of these factors to gain a deeper understanding of the role of psychological variables in neck discomfort among OWs.

Limitations and future directions

Future research should address the various limitations of our study. Firstly, the cross-sectional design impeded establishing a causal relationship between the identified characteristics and NP. Longitudinal studies are necessary to elucidate temporal associations and identify NP onset and chronicity predictors. Second, our reliance on self-reported data may have introduced subjectivity and recall bias in pain assessment. Future investigations could incorporate objective neck function and pain measures, such as pressure pain thresholds or range of motion assessments, to complement the self-reported data. Moreover, our study's single-institution scope may have limited our findings' generalizability to other settings within Saudi Arabia or the broader Middle Eastern region. Population-based surveys or multicenter studies would provide a more comprehensive understanding of NP prevalence and its associated factors in the region.

## Conclusions

Our findings showed that many participants experienced work absenteeism due to NP; however, only a minority sought medical attention, suggesting potential barriers to healthcare utilization. NP significantly affected life satisfaction and QALY, with a relatively small percentage of participants reporting high satisfaction with their health and sleep quality. Notably, individuals without a history of neck-related accidents are less likely to experience prolonged NP. While this study did not identify significant associations between NP duration and factors such as age, gender, or work hours, it emphasizes the necessity for tailored workplace interventions and improved access to medical services. Future research should employ a longitudinal design to elucidate causal relationships and investigate barriers to seeking medical attention. Despite limitations such as self-reported data and a single-institution setting, we believe this study contributes significantly to understanding NP in OWs.
